# “It is becoming increasingly difficult to find reviewers”—myths and facts about peer review

**DOI:** 10.1007/s00359-023-01642-w

**Published:** 2023-06-15

**Authors:** Günther K. H. Zupanc

**Affiliations:** Department of Biology, Northeastern University, Boston, MA 02115 USA

**Keywords:** Peer review, Editor, Invited reviewer, Research evaluation, Reviewer fatigue, Scientific publishing

## Abstract

A frequent complaint of editors of scientific journals is that it has become increasingly difficult to find reviewers for evaluating submitted manuscripts. Such claims are, most commonly, based on anecdotal evidence. To gain more insight grounded on empirical evidence, editorial data of manuscripts submitted for publication to the Journal of Comparative Physiology A between 2014 and 2021 were analyzed. No evidence was found that more invitations were necessary over time to get manuscripts reviewed; that the reviewer’s response time after invitation increased; that the number of reviewers who completed their reports, relative to the number of reviewers who had agreed to review a manuscript, decreased; and that the recommendation behavior of reviewers changed. The only significant trend observed was among reviewers who completed their reports later than agreed. The average number of days that these reviewers submitted their evaluations roughly doubled over the period analyzed. By contrast, neither the proportion of late vs. early reviews, nor the time for completing the reviews among the punctual reviewers, changed. Comparison with editorial data from other journals suggests that journals that serve a smaller community of readers and authors, and whose editors themselves contact potential reviewers, perform better in terms of reviewer recruitment and performance than journals that receive large numbers of submissions and use editorial assistants for sending invitations to potential reviewers.

## Introduction

When I talk to other editors, they frequently complain about increasing difficulties to find reviewers for evaluating submitted manuscripts. These comments are often supported by anecdotes like “I recently had to invite *n* potential reviewers to finally receive two review reports.” The largest *n* I heard in recent years was 40. No question, just the thought of having to come up with the names of 40 suitable reviewers is not something that even the best-networked editor looks forward to!

Although I haven’t seen numbers as large as 40 among the Editorial Board of the Journal of Comparative Physiology A, in some (rare?) instances it is, indeed, necessary to approach an unusually large number of potential reviewers. Recently, I had to invite 19 colleagues to receive two reviews for a manuscript. However, how well do ‘gut feeling’ and ‘anecdotal evidence’ reflect the actual situation at a given journal and at scientific journals in general? Has it really become more difficult over the last few years, including the years dominated by the COVID-19 crisis, to find reviewers who accept the editors’ invitation and (!) deliver their reports, hopefully within the agreed time frame? And along similar lines: Has the recommendation behavior of reviewers—whether they recommend rejection, acceptance, or revision of manuscripts—changed over these years?

To address some of the myths frequently expressed about peer review in scientific publishing, I analyzed data collected during editorial processing of manuscripts submitted to the Journal of Comparative Physiology A between 2014 and 2021. Like erroneous beliefs about reviewers suggested by authors (Zupanc 2022), the results debunk most of these myths at the Journal and argue for a more differentiated perspective in general. At the same time, the evidence presented might help authors and readers to better understand the review process that manuscripts undergo after submission to a scientific journal.

## Methodology

For the analysis, reviewer invitation data were collected over the period between January 1, 2014, and December 31, 2021. Throughout these 8 years, Editorial Manager (Aries Systems, North Andover, Massachusetts) was used by the Journal of Comparative Physiology A as its online platform for submission, peer review, and editorial processing of manuscripts. Since Editorial Manager had become the Journal’s management platform on July 1, 2013, editors were (presumably) familiar with its peer-review functions by January 1, 2014, when the analysis for the present Editorial started. On the other hand, as SNAPP (Springer Nature’s Article Processing Platform) replaced Editorial Manager in February 2022, and the peer-review functions of these two systems differ considerably, I decided to not extend the analysis beyond December 31, 2021, to avoid possible data distortion.

Statistical data for each of the 8 years were compiled using the Journal Accountability Report function in Editorial Manager. For statistical analysis, one-way ANOVA was conducted in IBM SPSS Statistics, version 28.0.0.0 (International Business Machines, Armonk, New York).

During the period of 2014–2021, 1097 new manuscripts (i.e., manuscripts with first receipt data during this time) were submitted to the Journal of Comparative Physiology A via Editorial Manager. During the same period, a total of 3776 reviewers were invited and 2304 reviews were completed. These numbers include reviews of original manuscripts and possible revised versions of the reviewed manuscripts. Revised manuscripts were, according to the standard editorial processing protocol, reviewed by the same reviewer(s) who evaluated the original submission. On the other hand, editors of the Journal of Comparative Physiology A avoided inviting reviewers excessively. This is indicated by the fact that the average reviewer completed just 2.2 reports over the entire 8 years analyzed.

### Myths versus facts

#### Myth 1: In recent years, the number of invitations necessary to get a manuscript reviewed has increased

**Fig. 1:**
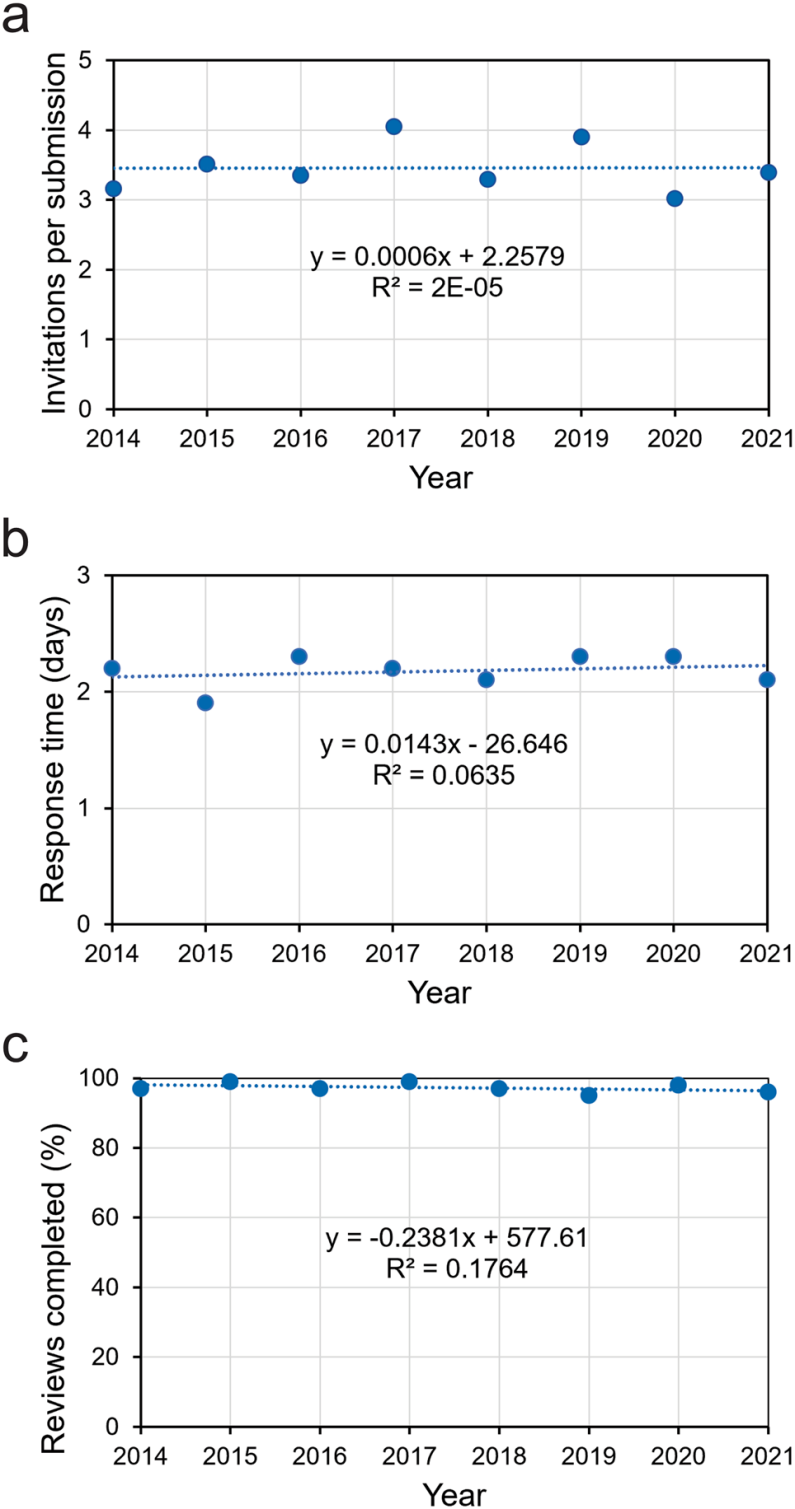
Recruitment and performance of invited reviewers. **a** Mean number of invitations per submission. **b** Mean time between sending out an invitation and receiving a response from potential reviewers. **c** Number of reviewers who completed their review reports, relative to the number of reviewers who had agreed to review a manuscript

To address this myth, I determined the number of invitations sent out per submission for every year between 2014 and 2021. This ratio fluctuated between 3.0 and 4.1 (Fig. 1a). A one-way ANOVA did not indicate a significant change over time (*p* = 0.992).

#### Myth 2: In recent years, it has taken longer and longer to receive a response to invitations from reviewers

To address this myth, I determined the mean time (in days) between sending out an invitation and receiving a response from potential reviewers. This response time has been remarkably constant, varying between 1.9 and 2.3 days during 2014–2021 (Fig. 1b). No significant change over time could be detected (*p* = 0.547, one-way ANOVA).

#### Myth 3: In recent years, the number of reviewers who completed their reviews has decreased

To address this myth, I used the number of reviewers who completed their review reports, relative to the number of reviewers who had agreed to review a manuscript. This fraction was notably high (0.95–0.99) and did not change significantly over the period examined (*p* = 0.240, one-way ANOVA) (Fig. 1c).

#### Myth 4: In recent years, reviewers have submitted their reports later and later

**Fig. 2:**
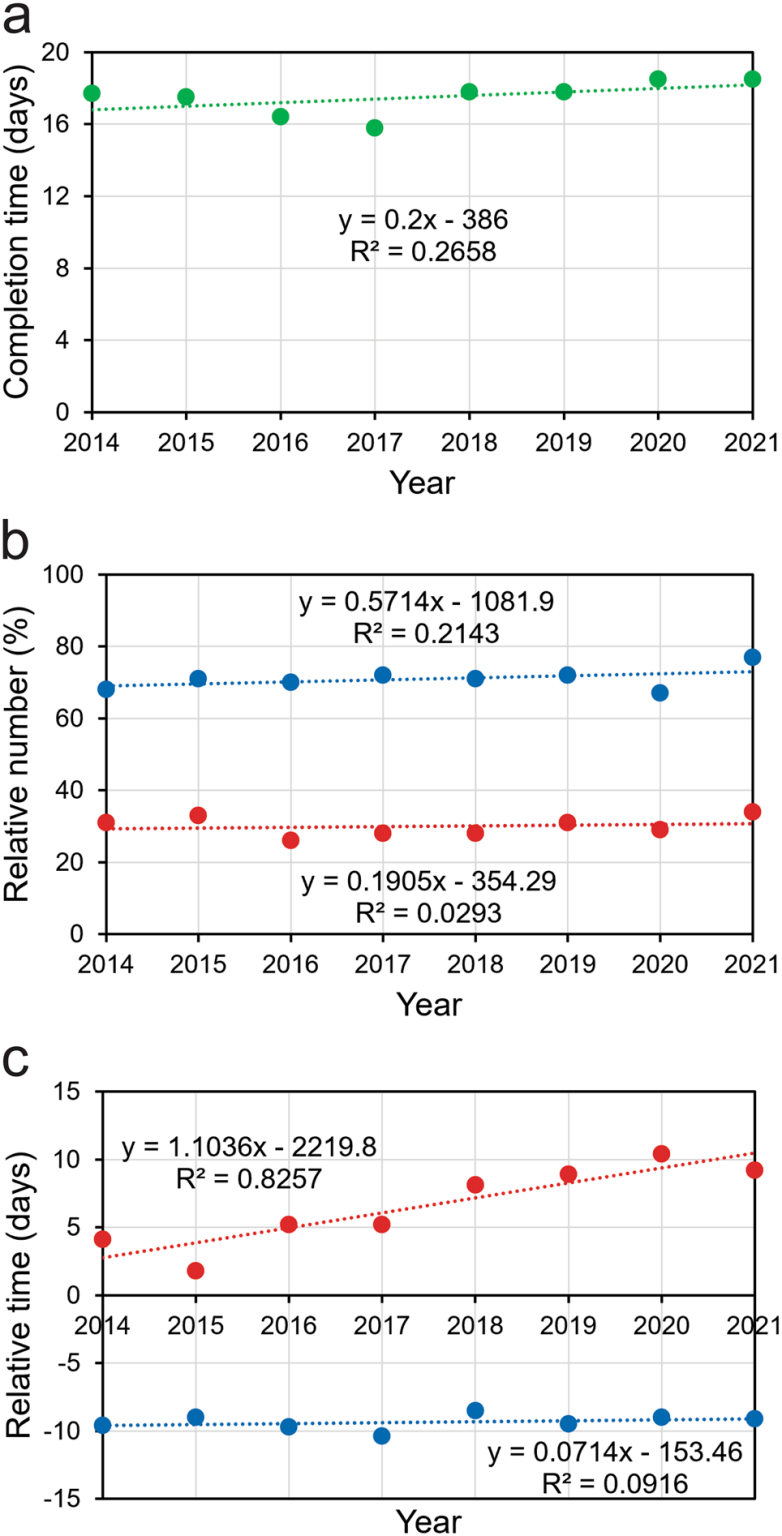
Time of submission of review reports, relative to agreed submission deadline. **a** Mean completion time. **b** Proportion of reviewers who submitted their reports earlier than agreed (blue circles and blue trendline) and reviewers who submitted their reports later than agreed (red circles and red trendline). **c** Mean number of days early reviewers submitted their reports earlier than agreed (negative numbers; blue circles and blue trendline) and late reviewers submitted their reports later than agreed (positive numbers; red circles and red trendline)

To address this myth, I first checked whether the mean number of days between the time reviewers had agreed to review a manuscript and the time they submitted their report changed between 2014 and 2021. No significant trend could be detected (*p* = 0.191, one-way ANOVA) (Fig. 2a).

However, closer examination of the completion time indicated that the situation was actually more complex because the punctuality behavior of the reviewers differed between two groups identified. Many of the reviewers (approximately two-thirds) submitted their reports earlier than agreed, whereas others (approximately one-third) were late. These fractions did not change significantly over the years (*p* = 0.266 and *p* = 0.716, respectively, one-way ANOVA) (Fig. 2b). However, different trends became evident in the number of days reviewers were early and the number of days reviewers submitted their reports late. Whereas the average number of days within which the early reviewers completed their reviews early did not change significantly between 2014 and 2021 (range: 8.5–10.4 days; *p* = 0.466, one-way ANOVA), the average number of days late reviewers submitted their reports late increased significantly over this period (range: 1.8–10.4 days; *p* = 0.002, one-way ANOVA) (Fig. 2c).

#### Myth 5: In recent years, reviewers have changed their recommendation behavior

**Fig. 3:**
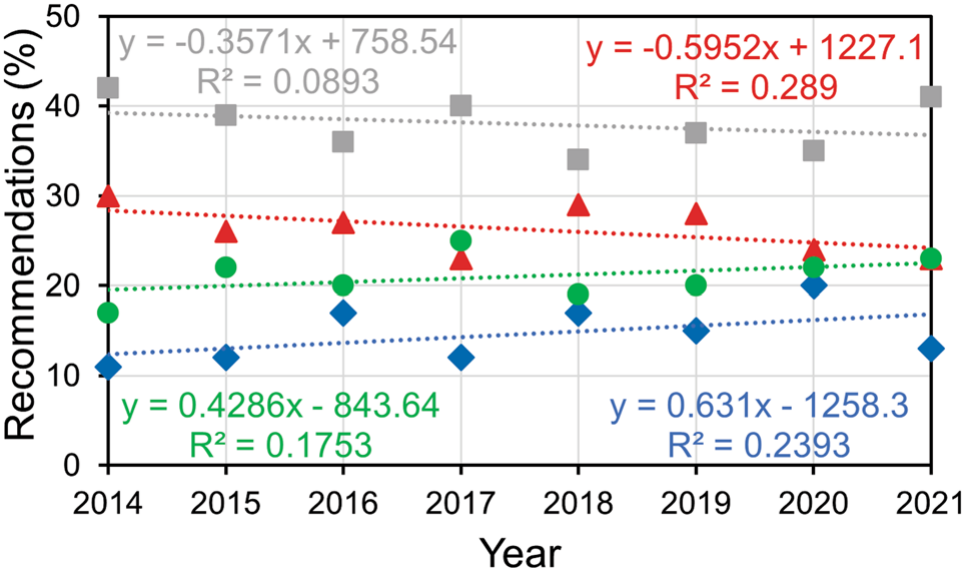
Recommendation behavior of reviewers. Percentage of reviewers who recommended rejection (blue diamonds and blue trendline), major revision (red triangles and red trendline), minor revision (gray squares and gray trendline), and acceptance (green circles and green trendline) of manuscripts

To address this myth, I examined possible trends over the years in terms of the following recommendations made by the reviewers: ‘reject,’ ‘major revision,’ ‘minor revision,’ and ‘accept’. Analysis showed that in none of these four categories did the reviewers change their recommendations significantly between 2014 and 2021 (*p* = 0.220, 0.161, 0.457, and 0.328, respectively, one-way ANOVA) (Fig. 3).

### Unusually large numbers of reviewer invitations in a few instances do not reflect the general situation of peer review

The findings of the above analysis are quite surprising, as they contradict the widely held belief that it is becoming harder to secure reviews for manuscripts submitted to scientific journals in general and to the Journal of Comparative Physiology A in particular. This is even more so, given that the last two years of the data collection—2020 and 2021—coincided with the beginning and peak of the COVID-19 pandemic when many people, including scientists, suffered from heightened stress and, presumably, reduced time availability for non-core tasks, such as reviewing manuscripts.

The trend that a portion (roughly one-third) of the reviews was completed later and later might, indeed, reflect such reduced time availability. At the same time, it is notable that the proportion of late reviews versus early reviews did not change; and the number of days that punctual reviewers submitted their reports early was highly constant over the period analyzed.

Apart from the significant trend in the time it took for late reviewers to complete their reviews, how can the mismatch between the widely perceived and generalized difficulty of finding reviewers for manuscripts, and the lack of empirical evidence in support of this notion at the Journal of Comparative Physiology A, be explained? Perhaps, the simplest explanation is that editors do not behave differently from ‘ordinary’ people when it comes to drawing unwarranted conclusions on the basis of one or a few instances. While it is true that in a few instances an excessive number of invitations is required to secure reviews for a manuscript, the claim that it has become more difficult to find reviewers in recent years is simply the result of a hasty generalization fallacy, like many other claims made in daily life.

### And what is the situation at other journals?

Despite important implications for peer review and scientific publishing in general, little empirical data have been published on recruitment and performance of reviewers at other journals. An analysis of six journals in the areas of ecology and evolution over the period of 2003–2015 found that the proportion of review invitations that led to submission of review reports had decreased steadily for four of these journals, yet no decline was evident for the other two journals (Fox et al. 2017). A similar retrospective analysis of editorial data over the period of 2010–2020 from the medical journal Acta Ophthalmologica (the official journal of the Nordic and Dutch Ophthalmological Societies and the European Association for Vision and Eye Research, which attracted an enormous number of submissions—2449—in 2020 alone) indicated an increase in the number of invitations needed to secure peer review (Bro and Hammarfelt 2022).

It is frequently assumed that reviewer fatigue caused by too many review requests is the driving force behind the increases seen in the number of reviewers declining the editors’ invitations. However, no convincing evidence has yet been presented in support of this claim. Further analysis of the data collected for Acta Ophthalmologica, and the four journals in ecology and evolution that had experienced a decline in the reviewers’ willingness to evaluate submissions, showed that the average number of reviews per individual reviewer, or the number of review requests per potential reviewer, had not increased over the examined time frame (Fox et al. 2017; Bro and Hammarfelt 2022). Increases in the number of submissions, as experienced by Acta Ophthalmologica, were offset by increases in rejections without peer review and by a larger reviewer database. This does not exclude the possibility that reviewers still received increased numbers of review requests from other journals. Alternatively, the reduced willingness of reviewers to participate in the review process, as found by some journals, may have its cause outside the scientific publication arena, for example in growing teaching or administrative workloads and/or in spending more time on the preparation of grant proposals, due to increased pressure to secure extramural funding.

### Why do some journals, including the Journal of Comparative Physiology A, do better than others?

The above empirical evidence, as limited as it may be, indicates that trends over recent years in recruitment and performance of reviewers differ among different journals. Yet, it is less clear what causes this difference. Fox et al. (2017) have speculated that one contributing factor is the method by which reviewers are contacted. In their analysis, three of the four ecology/evolution journals that showed a significant decline in positive reviewer response utilize editorial assistants who contact prospective reviewers on behalf of editors, whereas the policy of the two journals that exhibited no significant decline is that the editors themselves send invitations to the reviewers. As with the latter two journals, at the Journal for Comparative Physiology A it is the editors themselves that contact potential reviewers. In addition, Editorial Manager, which was used as a manuscript and editorial processing platform during the analyzed period, offers the option to modify the invitation letter to add, for example, a personal note. Editors frequently made use of this option.

Another factor that might play a role in the reviewers’ response is the size of the community that a journal is serving. Journals that receive well over 1000 submissions per year, such as the Acta Ophthalmologica, need to rely on large databases and on search algorithms to identify suitable reviewers whom the editors, in many cases, do not personally know. By contrast, the community served by the Journal of Comparative Physiology A is much smaller. Many of the potential reviewers who are invited to evaluate manuscripts are known personally to the editors, and a substantial portion of them have authored papers that have appeared in the Journal. Each member of the current Advisory Board—a main source for reviewer recruitment—has published in the Journal of Comparative Physiology A, and over 20% of them have authored 10 or more articles. This has created a sense of belonging to the loyal community of readers, authors, and reviewers. Nevertheless, the editors never take the service of the reviewers for granted. On behalf of the Editorial Board, I would like to express our gratitude to all of them for volunteering their time to advise us and to improve the quality of the articles that are submitted and eventually published.

## Data Availability

All data used for analysis are available from the author upon request.
